# EFFECTS OF INTERPERSONAL AND INTRAPERSONAL PROMOTION STRATEGIES ON FALLS: A PHYSICAL ACTIVITY TRIAL

**DOI:** 10.1093/geroni/igad104.1979

**Published:** 2023-12-21

**Authors:** Siobhan McMahon, Jean Wyman, Weihua Guan, Elizabeth Choma, Shannon Hayes, Qi Wang, Alexander Rothman, Beth Lewis

**Affiliations:** University of Minnesota, Minneapolis, Minnesota, United States; School of Nursing, University of Minnesota, Minneapolis, Minnesota, United States; University of Minnesota, Minneapolis, Minnesota, United States; Whitworth University, Spokane, Washington, United States; University of Minnesota - Twin Cities, Minneapolis, Minnesota, United States; University of Minnesota, Minneapolis, Minnesota, United States; University of Minnesota, Minneapolis, Minnesota, United States; University of Minnesota, Minneapolis, Minnesota, United States

## Abstract

Although fall-reducing physical activity (PA) interventions have promising effects on falls, little is known about the relative effects of different PA promotion strategies within such interventions. This trial assessed the effects of two promotion components within a PA intervention on participants’ falls 12 months post-intervention. The components included interpersonally-oriented (e.g., social support, social comparison) or intrapersonally-oriented (e.g., goal-setting, action planning) behavior change strategies informed by empirical evidence and theory. In this 2x2 factorial trial (Ready-Steady3 [RS3]),309 community-dwelling older adults (mean age 77+/-5) with > 1 fall risk factor were randomized to 1 of 4 intervention conditions. Factors represented random assignment (No, Yes) to interpersonal or intrapersonal components. All intervention conditions included the Otago Exercise Program adapted for small groups and a PA monitor (Fitbit) and were delivered in community settings over 8 weekly meetings, 90 minutes each. We assessed fall rates using participants’ documentation of falls on prospective monthly calendars. The main and interaction effects of interpersonal and intrapersonal components on fall rates were examined using negative binomial regression. The estimated fall incidence rates among participants assigned vs. those not assigned to the interpersonal component or the intrapersonal components, respectively, were:1.0 (+/-1.4) and 0.9 (+/-1.3), and 1.1 (+/-1.6) and 0.9 (+/-1.1) per person-year. Neither the main nor the interaction effects of either component were statistically significant, suggesting that interpersonal and intrapersonal components in RS3 PA interventions did not affect fall rates. Further investigation is warranted to identify which promotion strategies and dosages reduce falls and injuries in community-dwelling older adults.

